# EFFECT OF PROBIOTIC ORAL ADMINISTRATION ON SKIN WOUND HEALING IN RATS

**DOI:** 10.1590/0102-672020190001e1457

**Published:** 2019-12-09

**Authors:** Eliane TAGLIARI, Leticia Fuganti CAMPOS, Antonio Carlos CAMPOS, Thaís Andrade COSTA-CASAGRANDE, Lúcia de NORONHA

**Affiliations:** 1 Postgraduate Program in Clinical Surgery, Health Sciences Sector, Federal University of Paraná; Curitiba, PR, Brazil; 2 Department of Surgery, Health Sciences Sector, Federal University of Paraná; Curitiba, PR, Brazil; 3 Professional Master Program in Industrial Biotechnology, Positivo University; Curitiba, PR, Brazil; 4 Pathology Laboratory, Pontifical Catholic University of Paraná, Curitiba, PR, Brazil

**Keywords:** Probiotics, Wound healing, Rats, Administration, oral, Cicatrização, Administração oral, Probióticos, Ratos

## Abstract

**Background:**

Manipulating intestinal microbiota with probiotics might stimulate skin response. Understanding all stages of the healing process, as well as the gut-skin-healing response can improve the skin healing process.

**Aim:**

To evaluate the effect of perioperative oral administration of probiotics on the healing of skin wounds in rats.

**Methods:**

Seventy-two Wistar male adult rats were weighed and divided into two groups with 36 each, one control group (supplemented with oral maltodextrin 250 mg/day) and one probiotic group (supplemented with *Lactobacillus paracasei* LPC-37, *Bifidobacterium lactis* HN0019, *Lactobacillus rhamnosus* HN001, *Lactobacillus acidophilus* NCFM^®^ at a dose of 250 mg/day), both given orally daily for 15 days. The two groups were subsequently divided into three subgroups according to the moment of euthanasia: in the 3^rd^, 7^th^ and 10^th^ postoperative days.

**Results:**

There were no significant changes in weight in both groups. Wound contraction was faster in probiotic group when compared to the controls, resulting in smaller wound area in the 7^th^ postoperative day. As for histological aspects, the overall H&E score was lower in the probiotic group. The probiotic group showed increased fibrosis from 3^rd^ to the 7^th^ postoperative day. The type I collagen production was higher in the probiotic group at the 10^th^ postoperative day, and the type III collagen increased in the 7^th^.

**Conclusion:**

The perioperative use of orally administrated probiotic was associated with a faster reduction of the wound area in rats probably by reducing the inflammatory phase, accelerating the fibrosis process and the deposition of collagen.

## RESUMO

**Racional:**

Manipular a microbiota intestinal parece auxiliar na resposta cutânea. Entender todas as etapas do processo de cicatrização, bem como a resposta intestino-pele-cicatrização, pode ser ferramenta complementar no reparo cutâneo.

**Objetivo:**

Avaliar o efeito de suplementação perioperatória de probióticos via oral na cicatrização de feridas cutâneas excisionais em ratos.

**Métodos:**

Setenta e dois ratos adultos, machos Wistar, foram divididos em dois grupos de 36, sendo um de controle (suplementado com maltodextrina 250 mg/dia) e outro probiótico (suplementado com *Lactobacillus paracasei* LPC-37, *Bifidobacterium lactis* HN0019, *Lactobacillus Rhamnosus* HN001, *Lactobacillus Acidophilus* NCFM^®^, 250 mg/dia).

**Resultados:**

Não houve modificação significativa de peso em ambos os grupos. A contração da ferida foi mais rápida no grupo probiótico, quando comparada ao controle, resultando em menor área cruenta no sétimo dia do pós-operatório. Quanto aos aspectos histológicos, o escore geral do HE foi menor no grupo probiótico. O grupo probiótico apresentou maior fibrose do terceiro ao sétimo dias pós-operatórios. A produção de colágeno tipo I foi maior no grupo probiótico no décimo dia pós-operatório, e do tipo III maior no sétimo.

**Conclusão:**

O uso perioperatório do probiótico via oral foi associado à redução mais rápida da área cruenta da ferida cutânea em ratos, possivelmente por reduzir a fase inflamatória, acelerando a fibrose e o processo de deposição de colágeno.

## INTRODUCTION

The skin is a varied ecosystem composed of 1.8 m^2^ of tissue that covers the whole body, rich in folds, cutaneous attachments and contains a diverse microbiota^11^. Recently, advanced molecular analyses of the cutaneous microbiota revealed a great diversity and these vary according to its topographic location on the body^7^. Healing is a dynamic cellular process involving molecular and biochemical events aimed at tissue reconstitution^3^ ^,^ ^4^. Healing can be evaluated by clinical, mechanical, biochemical and histological parameters^3^ ^,^ ^4^. The microbiota of the skin also plays a key role in the immunological response and can interfere in the wound healing^20^. The perception of the skin as an ecosystem rich in living biological components and present in different locations explains the delicate balance between host and microorganisms. The skin microbiota is influenced by the intestinal microbiota and has also been shown to interact with the host symbiotically, modulating inflammation and the immune system, acting on the biotransformation of xenobiotics and the absorption of micronutrients, synthesizing vitamins, enzymes and proteins used by the host, fermenting energy substrates, providing resistance to pathogens and changing the amount of energy available in the diet^26^. The manipulation of the healing process with the use of probiotics has been studied both by topical application as well as by oral use. Probiotics are defined by the World Health Organization as “live microorganisms that when given in adequate amounts, confer a health benefit to the host^8^ ^,^ ^24^ ^,^ ^26^ ^,^ ^29^. Probiotics have been associated with improved healing of intestinal ulcers and healing of cutaneous wounds, among other actions already described in the literature^20^.

The objective of this study was to analyze the effect of the oral administration of probiotics on cutaneous healing in rats by macroscopic and histological aspects as well as by the deposition of collagen on the wound.

## METHODS

The study was part of the research on Tissue Healing of the Graduate Program in Surgery of the Federal University of Parana, Curitiba, Brazil. The animal experiments were carried out in accordance with the norms established by the Brazilian Federal Law No. 11,794, of October 8, 2008, Resolution 196/96 of the National Health Council, norms foreseen by the National Council of Control of Animal Experimentation (CONCEA) after approval of the Ethics Committee on the Use of Animals (CEUA) of the Positivo University (Opinion No. 294).

### Animals and probiotic administration

During the whole experiment the animals were packed in appropriate polypropylene boxes with wooden chips bed, receiving water and fed with Presence^®^-Purina ad libitum. Two animals were housed per box, kept in an air conditioned room at a constant temperature of 21° C, with humidity control and exposed to the brightness of 12 h of light a day, automatically controlled. A total of 72 adult male Wistar rats weighing +250 g obtained from the Positivo University laboratory were used. The rats were weighed and divided into two groups with 36 animals each, one control which received maltodextrin 250 mg/day) and one probiotic group supplemented with a probiotic Probiatop^®^ from FQM-FARMA compound that contained *Lactobacillus paracasei* LPC-37, *Bifidobacterium lactis* HN0019, *Lactobacillus rhamnosus* HN001, *Lactobacillus acidophilus* NCFM^®^, at the dose of 250 mg/day, which corresponds to the approximate dose of 200,000 to 210,000 CFU (colony forming units), administered orally once a day, starting five days before surgery until the euthanasia day, with the aid of a spatula^24^ mixed in cream cheese. The two groups were subsequently divided into three subgroups according to the moment of the euthanasia in the 3^rd^ postoperative (3PO) day; 7^th^ postoperative (7PO) day and 10^th^ postoperative (10PO) day, with 12 rats each.

### Surgical procedure

**FIGURE 1:**
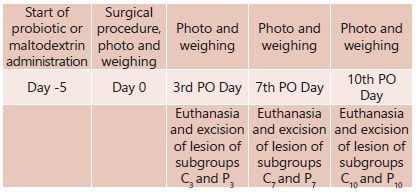
Study design

After five days of preoperative oral administration of either probiotics or maltodextrin, the rats were anesthetized and subjected to an excisional dorsal square wound, standardized by a mold measuring 2x2 cm. The anesthesia was via inhalation (isoflurane) and then maintained with an association of ketamine hydrochloride 80 mg/kg and hydrochloride of xylazine 10 mg/kg intramuscularly, being maintained under the inhalation effect of the anesthetic throughout the procedure. After recovery, they returned to their original cages receiving water and were allowed rat chow ad libitum. Liquid acetaminophen was used in a daily dose of 200 mg/kg/day orally, until the 4^th^ postoperative day. They were evaluated in 3PO; 7PO and 10PO days (Figure 1).

The wounds were photographed at a standard distance of 15 cm. The analysis and calculation of the areas, in square millimeters, were performed by the Image-Pro^®^ Plus software (version 4.5, Media Cybernetics, Rockville, Maryland, USA).

### Euthanasia and collection of materials

On the 3PO (C_3_ and P_3_), 7PO (C_7_ and P_7_) and 10PO (C_10_ and P_10_) days the rats were also submitted to euthanasia in a closed system with isoflurane. Immediately after death, the lesions were excised and the entire wound extension with a 1 cm margin of intact skin were included and stored with 10% formaldehyde in order to preserve their morphological structures for later histological study and collagen densitometry determination.

### Histological analysis

The pieces were cut into blocks in a rotating microtome, with cuts of five micrometers thickness. For each wound, a histological slide was made with 3-6 cuts. The sections were executed perpendicular to the surface of the dermis, in the central region and at the edges of the wound and submitted to dehydration and diaphanization in xylol and stained with H&E. The reading of the slides was performed under an Olympus BX40 optical microscope (Tokyo, Japan), with magnifications of 20x. The types and quantity of the predominant cells in the inflammatory reaction (neutrophils), the presence of interstitial edema and vascular congestion, and the degree of fibroblast, neovascularization and macrophage tissue formation were evaluated. The data were classified as accentuated (3), moderate (2) and discrete (1), according to the intensity in which they were found, and transformed into quantitative variables by assigning index to the histological findings. The presence of edema, congestion and polymorphonuclear cells were indicative of an acute inflammatory process, punctuating negatively, and the formation of fibroblasts, neovascularization and monocytes were findings that indicated a chronic inflammatory process, punctuating positively. After the assignment of the indices, these were added to a total final score for subsequent statistical comparison between the groups^25^.

### Collagen densitometry

Histological slides were stained with Picrosirius-red F3BA and photographed, were each image was captured under normal light and polarized light. Four fields per wound were selected for the slide readings, in a standardized way, two of the border and two of the central area of the wound, always from top to bottom. Images were transmitted from the ScopeA1^®^ microscope (Zeiss, Germany) connected to the AxioCam MRc (Zeiss, Germany) digital camera to an HP ZR2440W color monitor. The images were recorded by AxioVision 4.9 Software and were analyzed by the Image-Pro Plus® 4.5 software (Media Cybernetics, Rockville, Maryland, United States). In the RGB (Red, Green, Blue) system, the thicker and more strongly birefringent collagen fibers are colored with red-orange (type I or mature collagen), and the thinner, sparsely birefringent fibers were colored with shades of green (type III or immature collagen).

### Statistical analysis

The results were described by averages, medians, minimum values, maximum values and standard deviations. For the comparison between the groups, on each day of evaluation, the non-parametric Mann-Whitney test was used. The comparisons between the evaluation days, within the control and probiotic groups, were made using the Kruskal-Wallis non-parametric test. Values of p<0.05 indicated statistical significance. The data was organized in an Excel spreadsheet and analyzed with the IBM SPSS Statistics^®^ software, v.20.

## RESULTS

Throughout the experiment, there were no deaths. The body weight did not present significant changes in any group during the whole experiment (day -5 (3PO p=0.150; 7PO p=0.410; 10PO p=0.107), day 0 (3PO p=0.867, 7PO p=0.0851, 10PO p=0.185), day 3PO (3PO p=0.741; 7PO=0.599; 10PO p=0.629), day 7PO (7PO p=0.730; 10PO p=0.549) and 10PO (10PO p=0.937). 

### Histological analysis 

**FIGURE 2:**
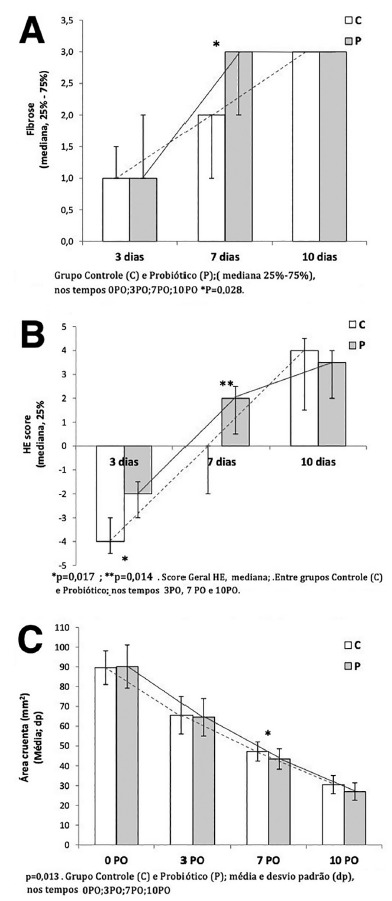
At times 3PO, 7PO and 10PO between Control (C) and Probiotic (P) groups: A) graph showing the amount of fibrosis; B) graph showing cell histological evolution; C) graph showing the wound tissue area (mm^2^), at times 3PO, 7PO and 10PO.

**FIGURE 3:**
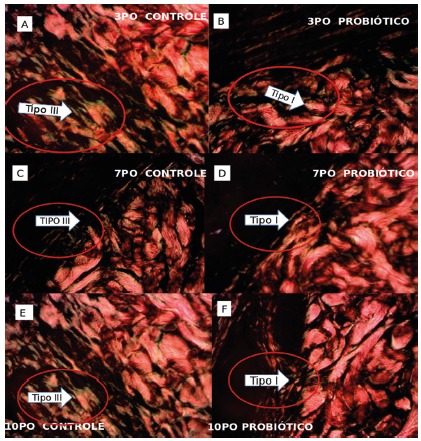
Histological evolution (edema, congestion, polymorphonuclear, fibrosis, neovascularization and monocytes) between control and probiotic: A and B) 3PO A=control, B=probiotic; C and D) 7PO C= control; D=probiotic); E and F) 10PO E=control and F=probiotic

In Figure 2A are shown the results for the histological indicators of the inflammatory reaction. For the edema, congestion and polymorphonuclear variables, there was no significant difference at any moment when the control and probiotic groups were compared. Fibrosis at the 7PO day was significantly higher in the probiotic group when compared to controls (p=0.028). For the neovascularization and monocyte variables, there were no significant differences between the groups. Also in Figure 2B it can be seen that the overall H&E score was better in the probiotic group when compared to the control in the 3PO (p=0.017) and 7PO (p=0.014). Figure 3 shows examples of the histological evolution (edema, congestion, polymorphonuclear, fibrosis, neovascularization and monocytes) between control and probiotic, at the 3PO, 7PO and 10PO days. It was observed that the probiotic group showed less edema, congestion and polymorphonuclears and the counting of the monocytes was equivalent.

Expressed in mean+standard deviation

Collagen type I (stained in red) and collagen type III (stained in green); Picrosirius-red F3BA (PSR), 20x

### Collagen analysis

**FIGURE 4:**
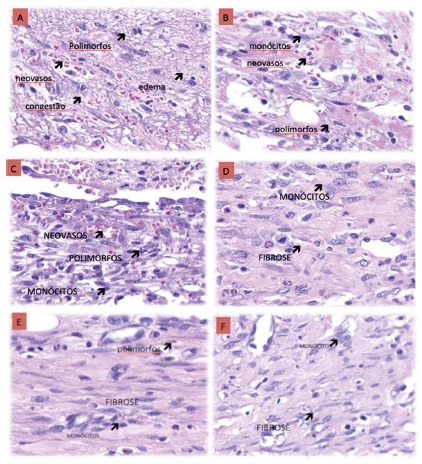
Example of histological evolution (edema, congestion, polymorphonuclears, fibrosis, neovascularization and monocytes) in both control and probiotic groups at the 3PO (A and B), 7PO (C and D) and 10PO (E and F) days

Table 1 shows the results of the evaluation of the wound deposition of Type I and Type III collagens. The type I was higher in the probiotic group on the 10PO (p=0.007) as compared to the control group. There was an increase in type III collagen in the 7PO (p=0.014) in the probiotic group when compared to the control group. In Figure 4 the distribution of type I and type III collagens is shown at times 3PO, 7PO and 10PO, for both the control and the probiotic groups.

**TABLE 1:** Evaluation of collagen I and collagen III (in the total area), expressed in mm^2^, in the control and probiotic groups, at times 3PO, 7PO and 10PO

Variable	Group	n	Mean ± standard deviation			p*(3^rd^ x 7^th^ x 10^th^)
			3PO day	7PO day	10PO day	
Collagen I						
Area (mm^2^)	Control	12	1.33 ± 0.88	0.96 ± 0.85	0.62 ± 0.61	0.313
	Probiotic	12	1.75 ± 0.66	0.30 ± 0.23	1.38 ± 0.73	<0.001
	p** (C x P)		0.266	0.028	0.007	
Collagen III						
Area (mm^2^)	Control	12	0.019 ± 0.014	0.018 ± 0.010	0.032 ± 0.027	0.598
	Probiotic	12	0.014 ± 0.013	0.029 ± 0.012	0.029 ± 0.028	0.023
	p** (C x P)		0.178	0.014	0.932	

### Wound tissue area

As described in Figure 2C, wound contraction was higher in the probiotic group in the 7PO when compared to the control, resulting in a smaller wound tissue area (43.4±5.2 vs. 47.2±4,9 mm^2^, p=0.013). As shown in Figure 4, the evolution of wound contraction was faster in the probiotic group as compared to controls.

## DISCUSSION

Probiotics and their metabolites are considered regulators of various biological functions, and their effects are being studied in the intestine-skin axis, controlling the healing of wounds^7^. Probiotics can be used topically or systemically (by the oral route). Many studies relate the benefits of applying probiotics topically, demonstrating improvement in wound healing by reducing bacterial load and increasing tissue repair in rodent wound models^16^. The systemic effects of probiotics promote the connection between the intestinal and the cutaneous microbiota, decrease inflammation, alter the composition of the microbiota in both sites and regulate the innate immune system^28^. The oral use improves the intestinal microbiota and the absorption of essential nutrients for the wound healing, such as vitamins, minerals and cofactors for the key enzymes involved in the regulation of cutaneous healing^16^ ^,^ ^28^.

Scotti et al.^23^ showed in their review that differences in the microbial composition of the intestine can affect the homeostasis of energy extraction, which may imply in gain or loss of weight in the host when supplemented with probiotic. In this study, there was no difference in weight on the days evaluated in any groups.

Wound healing is a highly dynamic process involving a complex sequence of cellular and biochemical events, ranging from an immediate response to skin cell damage and invasive microbial signals to inflammatory, angiogenic, and ultimately fibroplasia and scar formation^3^ ^,^ ^4^ ^,^ ^21^ ^,^ ^28^. The phenomena occur simultaneously, self-regulated and interfering with each other. It has been divided into three dynamic phases: inflammatory phase, proliferative phase and remodeling phase^4^ ^,^ ^5^ ^,^ ^29^. The interaction between inflammation, cell and humoral responses with intense cytokines production and liberation is fundamental for the healing process itself^21^. The present study describes a model of cutaneous excisional wound healing in rats in order to evaluate the effect of the oral administration of probiotic on skin wound healing. Macroscopic and microscopic evaluations of cutaneous wounds in rats were done in three different moments: the 3PO, 7PO, and 10PO days.

Neutrophils are the first cells recruited to the lesion area. Their main function is to protect the host from infection by combating invasive microorganisms and/or by removing cellular debris, as well as presenting antigens. However, activated neutrophils secrete bioactive substances, such as proteases and free radicals, which can lead to tissue damage if in excess^5^ ^,^ ^28^. The keratinocytes then migrate into the injured dermis and proliferate to form the granulation tissue which is intended to restore skin barrier function. Fibroblasts invade the clot and angiogenesis occurs, followed by tissue remodeling, controlled by fibroblasts that produce collagen and form the scar^5^ ^,^ ^17^.

Many monocyte and macrophage functions have been linked to the activation of Toll-like receptors (TLRs)^12^ ^,^ ^13^ ^,^ ^15^ ^,^ ^18^. The response to skin lesions in animals is triggered by molecular patterns associated with host-derived damage and the activation of inflammatory cells^13^. The findings of acute inflammatory process, such as interstitial edema and vascular congestion, are less closely linked to the process of cell proliferation, whereas the chronic inflammatory process is histologically related to the polymorphonuclear infiltrate, granulation tissue and fibrosis^25^. Fibrosis is defined as the interstitial fiber deposit that marks the onset of the scar^25^.

The proliferative phase, marked by the presence of fibrosis, was faster in the probiotic group on the 7PO day, when compared to the controls, resulting in a smaller wound tissue area at that moment. Probably the use of probiotic stimulated the collagen deposition and facilitated the fibrosis, improving the healing process. The amount of collagen on the 7PO day in the probiotic group was equivalent to that observed in controls on the 10PO day. When analyzing the H&E general score, it is expected that in the initial phase (3PO) the scores are negative, but in the probiotic group these were less negative. At 7 days (7PO), when the subacute phase starts, the probiotic group score was already positive, showing a faster resolution of the healing process in the probiotic group when compared to the control group. In the 10PO the probiotic group showed signs of stabilization of the chronic healing phase and signs of matrix remodeling.

The reduction of the wound area was faster in the probiotic group. By comparing wound area contraction, fibroblast proliferation and histological evolution it is clear that the group supplemented with probiotic had a better resolution of the healing process.

A possibly involved mechanism in this process is related to the role of TLRs^12^ ^,^ ^13^ ^,^ ^15^ ^,^ ^18^ ^,^ ^22^. External and internal epithelial coating tissues express TLRs, such as the skin and the gut. The commensal microbiota expresses antimicrobial peptides (AMPs)^13^ ^,^ ^15^. AMPs also stimulate and increase TLR pathways. They induce the production of chemokines with chemotactic activity and also modulate the function of dendritic cells and T-lymphocytes to promote wound healing and to maintain skin barrier homeostasis^12^ ^,^ ^13^ ^,^ ^15^ ^,^ ^18^. The expression of TLR in neutrophils, fibroblasts, monocytes and macrophages is essential for the healing response^18^ ^,^ ^22^. The appearance of TLR ligands indicates change in tissue integrity, which requires containment and repair. TLR-deficient animals were delayed in wound healing, especially in the neovascularization, re-epilation and fibrosis processes^18^. The role of TLRs in cutaneous healing has been heavily explored in scientific studies, especially in relation to scar fibrosis^12^ ^,^ ^13^ ^,^ ^15^ ^,^ ^18^ ^,^ ^19^ ^,^ ^22^. According to the results found in the histological markers, it is believed that these were probably related to the action of probiotics, via TLRs, on the cutaneous healing in rats.

The findings were also confirmed by the collagen deposition analysis. Type III collagen is rich in water, poorly polymerized and works as a filler. Type I is low in water, very polarized and its function is traction. The resistance of a scar is given by the amount of collagen deposited and by the way the fibers are deposited^10^ ^,^ ^22^. The healing process begins from the periphery to the center, boosting the organization of the fibers to remodel the collagen. The filling given by type III collagen to the scar area was greater and occurred on the 7PO in the probiotic group, but not in the control group, resulting in a greater production of collagen type I, which was greater in the probiotic group at the 10PO day. Poutahidis et al.^19^ evaluated the oral use of probiotic drink (*L. reuteri*) in mice, and the wound closure was marked by accelerated maturation of the granulation tissue and collagen deposition, what occurred from the sixth postoperative day in the group supplemented with *L. reueri* when compared to the controls supplemented with water, similar to our findings.

Modulating the microbiota using probiotics of different species involves a variety of possible mechanisms, including: a) competition with pathogenic bacteria by nutrients and binding sites in the host cell; b) inactivation of toxins and metabolites; c) production of antimicrobial substances that inhibit the growth of pathological microorganisms; d) stimulation/modulation of the host immune response, involving epithelial cells, dendritic cells and regulatory T- lymphocytes, both in the gastrointestinal tract and in the skin^6^ ^,^ ^19^.

Advanced studies done in animal models and also in humans showed the beneficial effects between gut bacteria and a healthier appearance of the skin. One of them, carried out by Levkovich et al.^24^, brought positive results with *Lactobacillus reuteri* supplementation in increasing dermal thickness and folliculogenesis, as well as potentiating sebum production and improving skin brightness^25^.

Heydari et al.^9^ studied the effect of probiotic (*L. plantarum*) on cutaneous wounds of rats, on days 1, 3, 7, 14 and 21PO, where the results showed an earlier acute phase, with a lower total number of neutrophils in the 3PO and reduction of the wound area, probably by reducing inflammation and the released growth factors, which may have helped to achieve an earlier re-epithelization. These findings corroborate the results presented here.

Probiotics also participate in the improvement of skin differentiation and keratinization process, in the modulation of the cutaneous immune response and in the process of cutaneous healing^19^. Probiotics given orally result in increased Treg Foxp3+ cells in the lymph nodes of the skin, positively regulating the expression of IL10, decreasing tissue damage at the border of the wound and reducing inflammation in the murine model^1^.

## CONCLUSION

The perioperative use of orally administrated probiotic was associated with a faster reduction of the wound area in rats probably by reducing the inflammatory phase, accelerating the fibrosis process and the deposition of collagen.
